# Supplementary material for: Low-Dose Eribulin Promotes NK Cell-Mediated Therapeutic Efficacy in Bladder Cancer
Source: Cancers (Basel). 2024 Nov 19;16(22):3875. doi: 10.3390/cancers16223875 (PMC11592921; doi:10.3390/cancers16223875)
Supplement: Supplementary file 1 [file cancers-16-03875-s001.zip › cancers-3271599-supplementary.pdf]

Supplementary Figures

Supplementary Figure 1. Eribulin treatment decreases the expression of exhaustion markers on NK cells *in vivo*

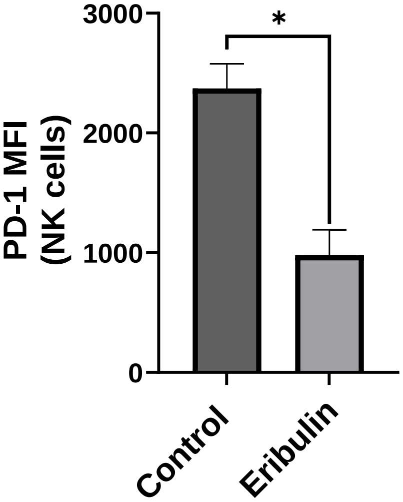

Supplementary Figure S1. Eribulin treatment decreases the expression of exhaustion markers on NK cells *in vivo* C57BL/6 mice were challenged by intravesical instillation of MB49 murine BCa cells and treated with 1.8 mg/kg of eribulin or 100 µL of DPBS by intravesical instillation weekly for four weeks beginning on day 1. Shown is the expression of PD-1 on tumor infiltrating NK cells from one representative results of two independent experiments (n=5 per group).

Supplementary Figure S2. NK cell depletion abolishes the anti-tumor effects of eribulin treatment in bladder cancer

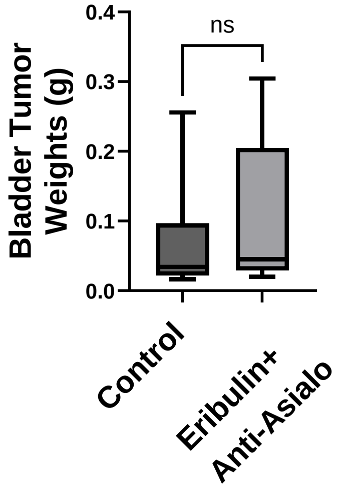

Supplementary Figure S2. NK cell depletion abolishes the anti-tumor effects of eribulin treatment in bladder cancer C57BL/6 mice were challenged by intravesical instillation of MB49 murine BCa cells and treated with 1.8 mg/kg of eribulin or 100 µL of DPBS by intravesical instillation weekly for four weeks beginning on day 1. NK cells were depleted by intraperitoneal injection of anti-Asialo GM-1, weekly starting 3 days prior to orthotopic challenge. On day 20, bladders were excised, and the bladder weight was recorded prior to digestion (n=5 per group). Ns: not significant.

Supplementary Figure S3. Eribulin treatment reduces tumor burden in humanized murine BCa model

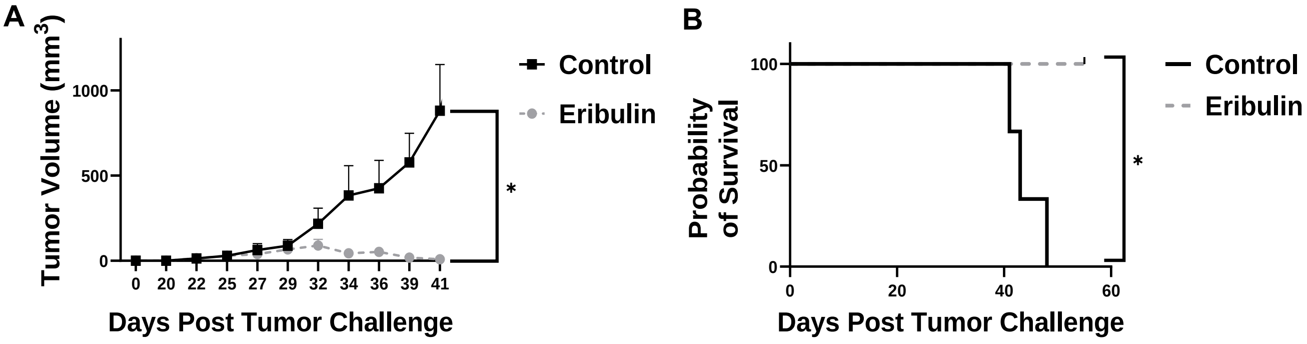

Supplementary Figure S3. Eribulin treatment reduces tumor burden in humanized murine BCa model NSG mice were injected subcutaneously with NK92.MI and UMUC3 BCa cells in a 3:1 ratio and treated with 1 mg/kg of eribulin by weekly intratumoral injection once tumors reached 100 mm^3 (approximately day 20). Tumor volumes were measured three times a week and mice were euthanized once tumor volumes reached 1500 mm^3 (**A**). *$p<0.05$ by a two-way ANOVA test. (**B**) Kaplan-Meier curve of NSG mice based on days post challenge until tumor volume reached 1500 mm^3. *$p<0.05$ by log-rank (Mantel-cox) test.

Supplementary Figure S4. Eribulin treatment significantly alters the RNA expression of genes involved in NK cell functionality

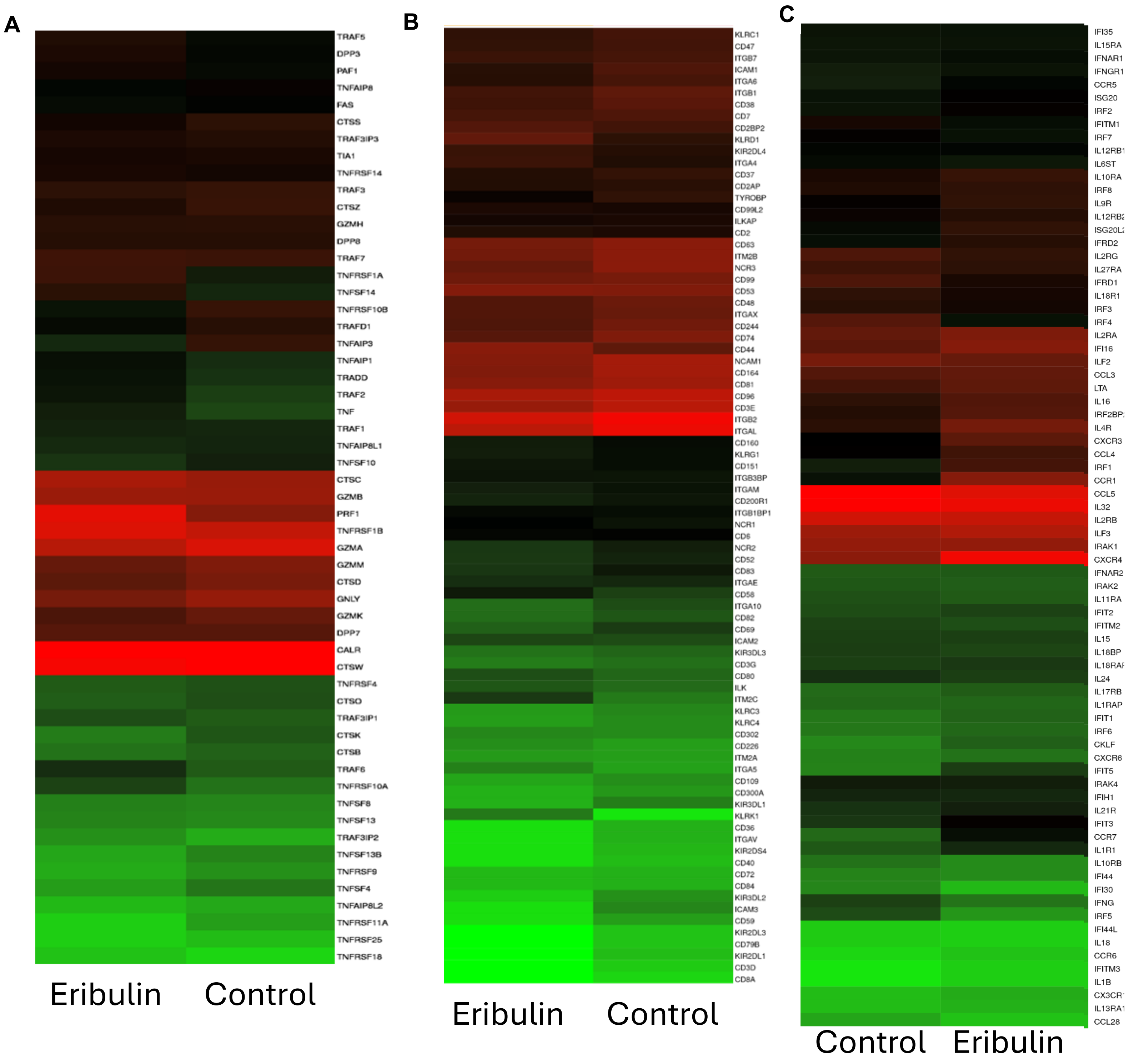

D

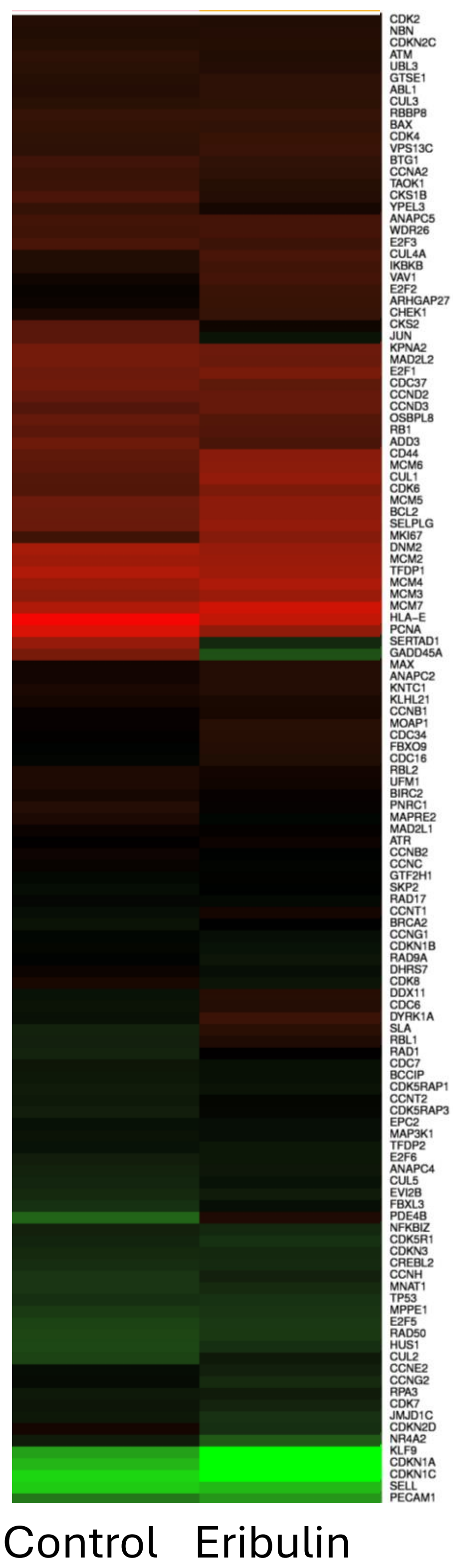
CDK2
NBN
CDKN2C
ATM
UBL3
GTSE1
ABL1
CUL3
RBBP8
BAX
CDK4
VPS13C
BTG1
CCNA2
TAOK1
CKS1B
YPEL3
ANAPC5
WDR26
E2F3
CUL4A
IKBKB
VAV1
E2F2
ARHGAP27
CHEK1
CKS2
JUN
KPNA2
MAD2L2
E2F1
CDC37
CCND2
CCND3
OSBPL8
RB1
ADD3
CD44
MCM6
CUL1
CDK6
MCM5
BCL2
SELPLG
MKI67
DNM2
MCM2
TFDP1
MCM4
MCM3
MCM7
HLA-E
PCNA
SERTAD1
GADD45A
MAX
ANAPC2
KNTC1
KLHL21
CCNB1
MOAP1
CDC34
FBXO9
CDC16
RBL2
UFM1
BIRC2
PNRC1
MAPRE2
MAD2L1
ATR
CCNB2
CCNC
GTF2H1
SKP2
RAD17
CCNT1
BRCA2
CCNG1
CDKN1B
RAD9A
DHRS7
CDK8
DDX11
CDC6
DYRK1A
SLA
RBL1
RAD1
CDC7
BCCIP
CDK5RAP1
CCNT2
CDK5RAP3
EPC2
MAP3K1
TFDP2
E2F6
ANAPC4
CUL5
EVI2B
FBXL3
PDE4B
NFKBIZ
CDK5R1
CDKN3
CREBL2
CCNH
MNAT1
TP53
MPPE1
E2F5
RAD50
HUS1
CUL2
CCNE2
CCNG2
RPA3
CDK7
JMJD1C
CDKN2D
NR4A2
KLF9
CDKN1A
CDKN1C
SELL
PECAM1
Control
Eribulin

E

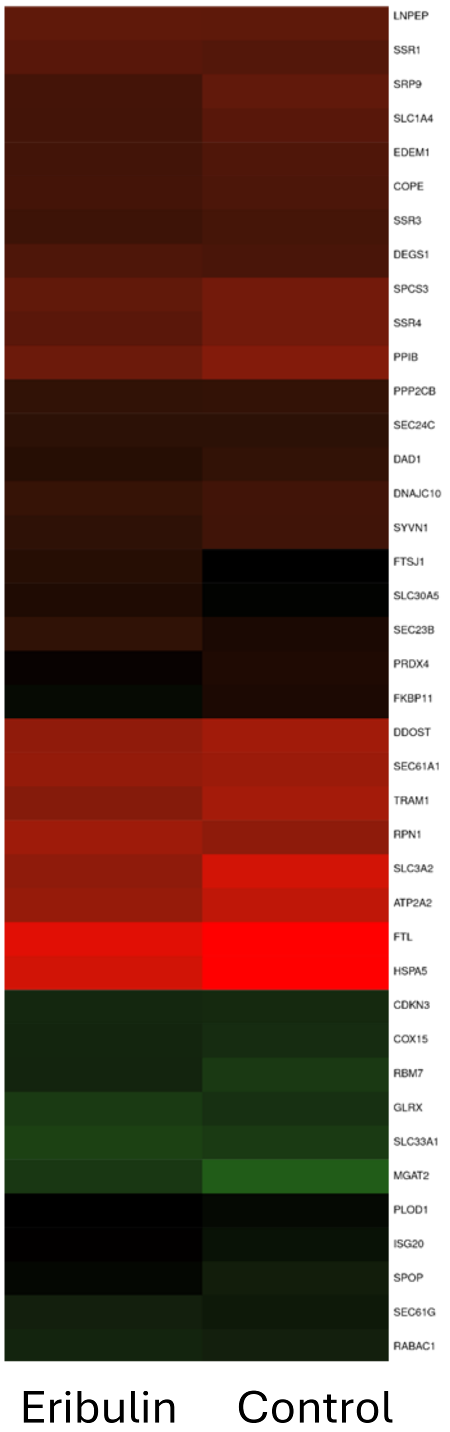
LNPEP
SSR1
SRP9
SLC1A4
EDEM1
COPE
SSR3
DEGS1
SPCS3
SSR4
PPIB
PPP2CB
SEC24C
DAD1
DNAJC10
SYVN1
FTSJ1
SLC30A5
SEC23B
PRDX4
FKBP11
DDOST
SEC61A1
TRAM1
RPN1
SLC3A2
ATP2A2
FTL
HSPA5
CDKN3
COX15
RBM7
GLRX
SLC33A1
MGAT2
PLOD1
ISG20
SPOP
SEC61G
RABAC1
Eribulin
Control

Supplementary Figure S4. Eribulin treatment significantly alters the RNA expression of genes involved in NK cell functionality Heatmap of bulk RNAseq data of NK92 cells treated with 100 nM of eribulin for 3 hours. Genes related to (**A**) cytotoxicity, (**B**) adhesion, (**C**) secreted cytokines, (**D**) cell cycle, (**E**) and quiescence.

Supplementary Figure S5. Subset of tumor infiltrating NK cells display a unique phenotype not seen in peripheral blood NK cells

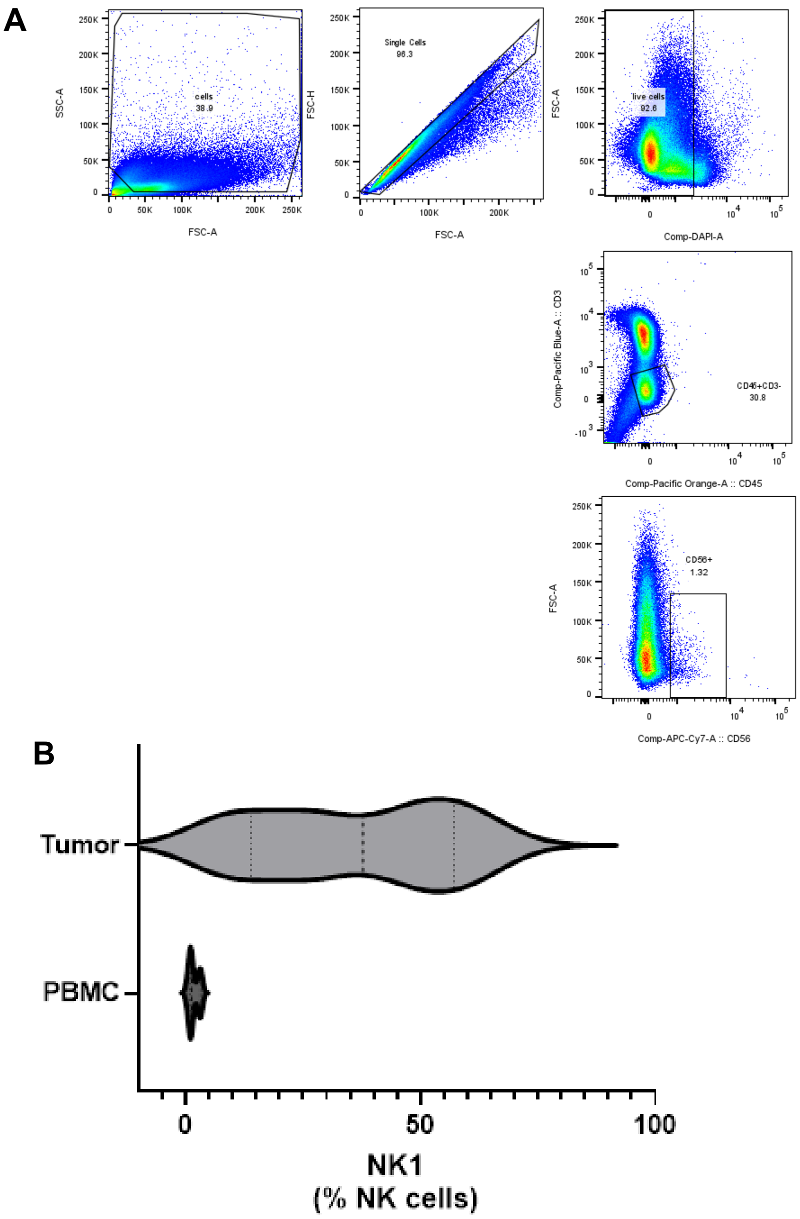

Supplementary Figure S5. Subset of tumor infiltrating NK cells display a unique phenotype not seen in peripheral blood NK cells Human peripheral blood and tumor samples were stained and analyzed by flow cytometry. **(A)** A representative gating strategy used to identify NK cell subsets in human PBMC samples. The population of doublets was first excluded, followed by selecting living cells (FVD$^-$). NK cells, defined as CD56$^+$ cells, were gated from the total CD45$^+$CD3$^-$ population. **(B)** Shown is the proportion of peripheral or tumor-infiltrating NK cells that are CD49a$^+$CD103$^+$. Values were based on pooled data from 3 patients (PBMC) and 4 patients (Tumor).

Supplementary Figure S6. Eribulin treatment influences TGFβ signaling related genes in NK cells

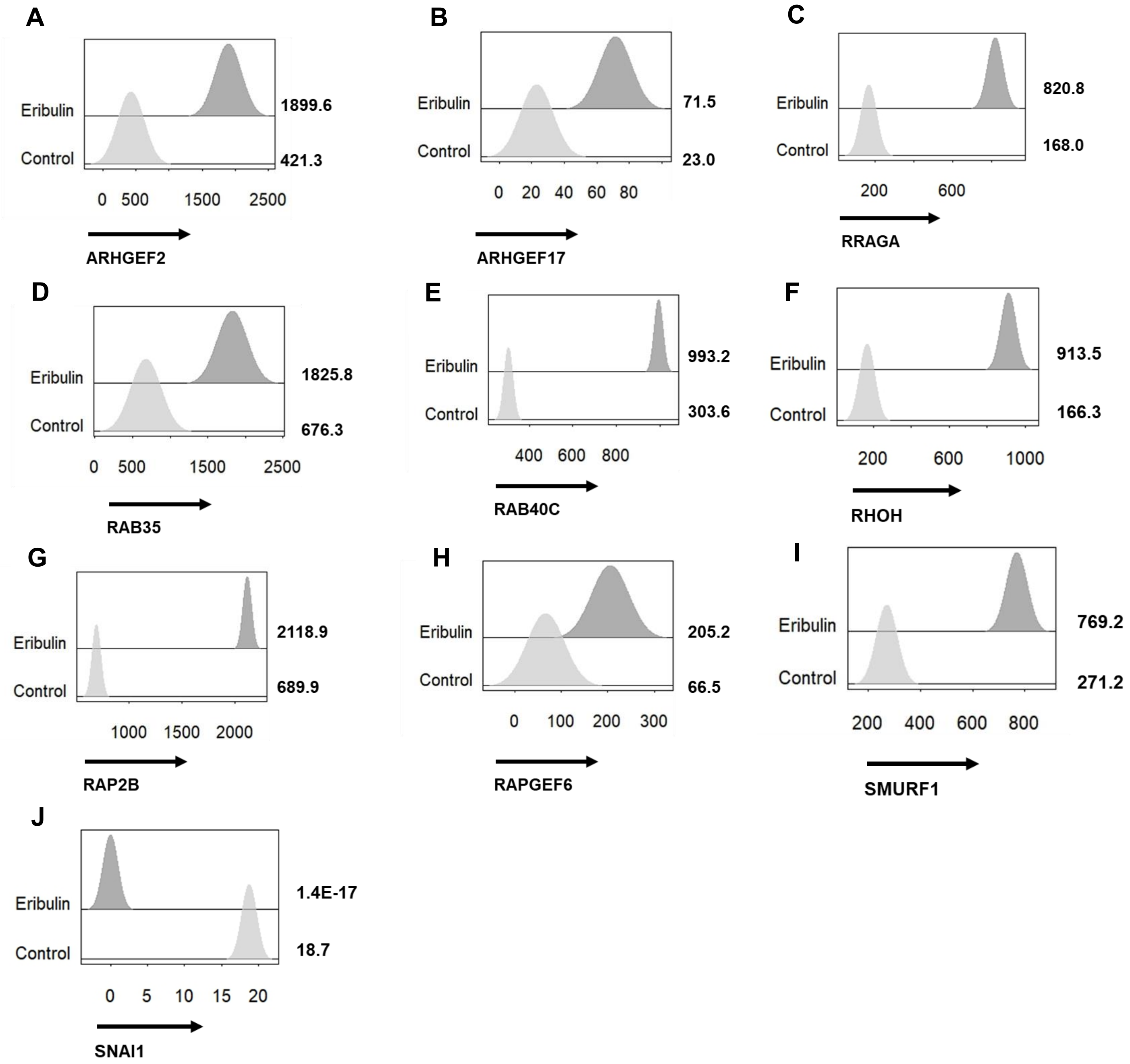

Supplementary Figure S6 Eribulin treatment influences TGFβ signaling related genes in NK cells

For bulk RNA sequencing, NK92 cells were treated with 100 nM of eribulin for 3 hours before extraction of RNA. Shown are the FPKM of (**A**) *ARHGEF2,* (**B**) *ARHGEF17,* (**C**) *RRAGA,* (**D**) *RAB35,* (**E**) *RAB40C,* (**F**) *RHOH,* (**G**) *RAP2B,* (**H**) *RAPGEF6,* (**I**) *SMURF1,* and (**J**) *SNAI1* illustrated as histograms. Differential analysis of illustrated genes was found to be significant, with p-values <0.05.
